# Anorectal Malignant Melanoma: Diagnostic Pitfalls and Prognostic Insights from a Single-Center Retrospective Analysis

**DOI:** 10.3390/diagnostics15162086

**Published:** 2025-08-20

**Authors:** Emre Hafızoğlu, Murat Bardakçı, Yakup Ergun, Irfan Karahan, Doğan Bayram, Fahriye Tugba Kos, Efnan Algın, Oznur Bal, Dogan Uncu

**Affiliations:** 1 Department of Medical Oncology, Afyonkarahisar State Hospital, 03100 Afyonkarahisar, Turkey; 2 Department of Medical Oncology, Gazi Yasargil Training and Research Hospital, 21100 Diyarbakır, Turkey; dr.muratbardakci@hotmail.com; 3 Department of Medical Oncology, Bower Hospital, 21100 Diyarbakır, Turkey; dr.yakupergun@gmail.com; 4 Department of Medical Oncology, Ankara Bilkent City Hospital, 06800 Ankara, Turkey; irfan_karahan@yahoo.com (I.K.); tugbasan@yahoo.com (F.T.K.); efnanalgin@gmail.com (E.A.); dr_ozn@yahoo.com (O.B.); doganuncu@yahoo.com (D.U.); 5 Department of Medical Oncology, Gülhane Training and Research Hospital, 06010 Ankara, Turkey; drdoganb@gmail.com

**Keywords:** anorectal malignant melanoma, prognosis, diagnostic delay

## Abstract

**Background and Objectives:** Anorectal malignant melanoma (ARMM) is a rare and aggressive mucosal melanoma with a poor prognosis. Due to its rarity and nonspecific clinical presentation, diagnosis is often delayed, and prognostic data remain limited. **Methods:** In this retrospective study, 17 patients diagnosed with ARMM were identified from a cohort of 404 malignant melanoma cases treated at our center; however, only 14 patients with complete clinical and pathological data were included in the final analysis. Demographic, clinical, and histopathological data were collected. Disease stage, treatment modalities, and survival outcomes were analyzed. Event-free survival (EFS) and overall survival (OS) were calculated, and potential prognostic factors were evaluated using univariate and multivariate analyses. **Results**: The mean age at diagnosis was 58 ± 12 years, and six patients (42.9%) were female. The median follow-up duration was 13.3 months, and the median OS was 12.6 months. Six patients (42.9%) were initially misdiagnosed due to overlapping symptoms with benign anorectal conditions. At presentation, seven patients had localized disease, while six had distant metastases. Univariate analysis identified male sex and liver metastasis as adverse prognostic factors for OS; however, these associations were not statistically significant in multivariate analysis. **Conclusions:** ARMM is associated with poor survival outcomes, and liver metastasis and male sex may be linked to worse prognosis. Diagnostic delay is common due to nonspecific symptoms and frequent initial misdiagnosis, highlighting the need for increased clinical awareness. Further large-scale studies are warranted to better define prognostic markers and optimize management strategies.

## 1. Introduction

Anorectal malignant melanoma (ARMM) is a rare and aggressive subtype of mucosal melanoma, accounting for approximately 0.05% of all colorectal malignancies, 1% of anal canal cancers, and only 0.3–1% of all melanomas. ARMM typically arises from melanocytes located in the transitional epithelium of the anorectal junction, though it can also originate from the anal verge, rectal mucosa, or anal canal. The anorectal region is the third most frequent site of mucosal melanomas after the head and neck and the female genital tract [1,2,3,4,5].

Clinically, ARMM often mimics benign anorectal disorders such as hemorrhoids or polyps, with common symptoms including rectal bleeding, anorectal pain, mass sensation, and altered bowel habits. Due to this nonspecific presentation, diagnosis is frequently delayed, and the majority of patients are diagnosed at locally advanced or metastatic stages, with frequent distant metastases to the liver, lungs, brain, and bone [5,6].

Histologically, ARMM can be challenging to distinguish from poorly differentiated carcinomas or lymphomas, particularly in amelanotic variants. Therefore, immunohistochemistry is crucial for diagnosis, with melanocytic markers such as S100 (soluble in 100% ammonium sulfate), HMB-45 (human melanoma black-45), and Melan-A (melanoma antigen recognized by T-cells 1) playing a central role [7,8]. Radiologic evaluation—including contrast-enhanced computed tomography (CT), magnetic resonance imaging (MRI), positron emission tomography–computed tomography (PET-CT), and endoscopic ultrasound (EUS)—complements the diagnostic process and aids in staging and treatment planning [9,10].

Due to the lack of a specific TNM classification system for ARMM, the Ballantyne staging system—originally developed for mucosal melanomas of the head and neck—has also been applied in some ARMM studies. This system categorizes the disease into three clinical stages: Stage I (localized disease), Stage II (regional lymph node involvement), and Stage III (distant metastasis). To improve prognostic accuracy, Falch et al. proposed a four-tier staging system incorporating tumor invasion depth [11,12,13].

Currently, surgical resection remains the cornerstone of treatment. However, the optimal surgical approach—wide local excision (WLE) versus abdominoperineal resection (APR)—remains controversial, as survival outcomes appear comparable [10,11,12,13]. Although chemotherapy, radiotherapy, and immunotherapy have been utilized in various settings, no standardized systemic treatment guidelines exist for ARMM [8,14,15,16].

Given the rarity of ARMM, available data on its clinicopathologic features and prognostic indicators remain limited. In this retrospective single-center study of 14 patients with primary ARMM, we aimed to highlight the clinical and diagnostic challenges of this aggressive malignancy, particularly the frequent delays and misdiagnoses due to its nonspecific presentation. Guided by the principle that “we cannot find what we are not looking for,” we sought to draw attention to the fact that ARMM is often overlooked in the differential diagnosis of colorectal diseases. By sharing our data, we aim to raise clinical awareness and contribute to earlier recognition and more accurate diagnosis of ARMM.

## 2. Materials and Methods

### 2.1. Patients and Research Design

We retrospectively reviewed the medical records of adult patients diagnosed with malignant melanoma between January 2002 and March 2023. Data were extracted from institutional electronic medical records and tumor registries. Among a total of 17 patients diagnosed with primary ARMM, 3 were excluded due to incomplete data, and 14 patients were included in the final analysis. The majority of patients were referred to our tertiary oncology department after histopathologic confirmation of ARMM, most often from secondary or non-tertiary hospitals. All surgical and pathological information was obtained directly from operative and pathology reports prepared by board-certified colorectal surgeons and pathologists at our institution or at referring centers. Incisional biopsy refers to a full-thickness biopsy of the primary lesion performed by either a colorectal surgeon or a gastroenterologist during endoscopy. A definitive diagnosis of ARMM was established by histopathological evaluation and positive immunohistochemical staining for S-100, HMB-45, and Melan-A.

We adopted the three-tier clinical staging system for ARMM: Stage I, Stage II, and Stage III. Information on tumor invasion depth, which is required for applying the Falch et al. four-tier staging system, was not available in our retrospective dataset and thus this system could not be utilized in the analysis. Collected variables included patient sex, age, date of diagnosis, Eastern Cooperative Oncology Group performance status (ECOG PS), tumor location, initial symptoms, tumor characteristics, clinical stage, histopathological features, BRAF (v-Raf murine sarcoma viral oncogene homolog B1) mutation status, presence of nodal and distant metastasis, surgical procedures, and systemic treatment modalities. Additionally, treatment outcomes were recorded. All patients were censored at the last follow-up or date of death, and only survival outcomes and prognostic factors of ARMM were evaluated.

Initial evaluations included physical examination, rectosigmoidoscopy, incisional biopsy, and radiologic imaging (thoracoabdominal CT and 18F-FDG PET/CT). EUS was not performed in any patient due to lack of availability in some referring centers and was not performed at our institution during the study period. Each patient underwent one or more treatment modalities, including WLE, APR, radiotherapy, chemotherapy, or immunotherapy. All APR and WLE procedures were performed either in our institution or at referring centers, with operative and pathology reports reviewed for details. Follow-up assessments were conducted every three months for two years and included digital rectal examination and thoracoabdominal imaging.

### 2.2. Statistical Analysis

Continuous variables were reported as mean ± standard deviation (SD) or median (range), while categorical variables were expressed as frequencies and percentages. Median follow-up time was calculated from diagnosis to last contact or death; event-free survival (EFS) from diagnosis to first documented recurrence, progression, or death; and overall survival (OS) from diagnosis to death from any cause.

Due to the limited cohort size (*n* = 14), several statistical adjustments were made to preserve the robustness and interpretability of the analyses. Certain variables were grouped to increase statistical power and prevent overfitting in the multivariate model. Age was dichotomized at the median (≤58 vs. >58 years), tumor location was classified as anorectal junction versus rectum/anal canal, and disease stage was grouped as Stage I versus Stage II–III. These groupings were essential to ensure stable hazard ratio estimation and align with practical clinical decision-making frameworks.

Kaplan–Meier survival analysis was used to estimate OS, and group comparisons were performed using the log-rank test. Variables with a *p*-value < 0.05 in univariate analysis were entered into a Cox proportional hazards regression model to identify independent predictors of OS. To ensure the validity of the model, multicollinearity among the independent variables was assessed using variance inflation factors (VIF <2) and correlation matrix diagnostics. No substantial collinearity was identified, and therefore, variables with *p*-values < 0.05 in univariate analysis, along with clinically meaningful variables, were included in the multivariate Cox proportional hazard model. This approach prioritized parsimony and robustness in line with best practices for statistical modeling in rare cancers. All tests were two-sided, and a *p*-value < 0.05 was considered statistically significant. Data analysis was performed using SPSS version 22.0 for Windows (SPSS Inc., Chicago, IL, USA).

### 2.3. Ethical Considerations

This study was conducted in accordance with the ethical principles outlined in the Declaration of Helsinki and approved by the Clinical Research Ethics Committee No. 1 of Ankara City Hospital (Protocol Code: E1-23-4294; Date of Approval: 15 November 2023).

## 3. Results

### 3.1. Baseline Characteristics

A total of 404 patients with malignant melanoma were treated at our center. Of these, 87% had cutaneous melanomas, while 13% had non-cutaneous melanomas. Among non-cutaneous cases, 57% originated in the uveal tract, 10% in the genitourinary tract, and 32% (*n* = 17) were diagnosed with ARMM. Due to incomplete medical records, 3 patients were excluded, and the final analysis included 14 cases.

The mean age at diagnosis was 58 ± 12 years. Eight patients (57.1%) were male and six (42.9%) were female, with a male-to-female ratio of 1.33:1. None of the patients had a family history of melanoma, and all had an ECOG PS of 0–1 at the time of diagnosis. The most common primary sites were the anal canal (42.9%) and the anorectal junction (42.9%), followed by the rectum (14.3%). Rectal bleeding was the most frequent presenting symptom (71.4%), followed by weight loss (42.9%), anorectal mass (35.7%), altered bowel habits (35.7%), and perianal pain (14.3%).

All patients presented to our oncology department after a histopathological diagnosis of ARMM had been established, and diagnostic delays were determined from patient histories. In our cohort, misdiagnoses and diagnostic delays occurred primarily at referring centers, prior to presentation to our institution. Six patients (42.9%) were initially misdiagnosed, two with hemorrhoids (14.3%), one with a rectal polyp (7.1%), and three (21.4%) had normal colonoscopic findings, leading to a median diagnostic delay of 5.3 months (range: 3–10 months). Notably, the majority of these patients were ultimately diagnosed at either locally advanced or metastatic stages. Despite undergoing endoscopic evaluation, the tumor was interpreted as polypoid in five patients (35.7%) and as a luminal mass in nine patients (64.3%). These nonspecific endoscopic appearances may have contributed to the misdiagnoses and highlight the diagnostic ambiguity of ARMM in its early stages.

All patients underwent CT and colonoscopy; MRI and PET-CT were performed in 12 (85.7%) and 13 (92.9%) patients, respectively. Endoscopic evaluation revealed a polypoid mass in five (35.7%) patients and a luminal mass in nine (64.3%). None of the patients underwent endoscopic ultrasound.

Immunohistochemically, all cases were positive for S-100, HMB-45, and Melan-A. Histologically, two tumors (14.3%) were amelanotic, three (21.4%) were melanotic, and nine (64.3%) were not specified. Pigmentation status was not reported in nine cases—mostly diagnosed in referring centers—limiting our ability to draw definitive conclusions regarding its prevalence and impact in this cohort. Ulceration was reported in four patients (28.6%), absent in four (28.6%), and unknown in six (42.9%). BRAF mutation testing was performed in 12 patients (85.7%) and was negative in all cases.

At diagnosis, seven patients (50%) had Stage I disease, one (7.1%) had Stage II, and six (42.9%) had Stage III. The liver (35.7%) and lungs (21.5%) were the most frequently involved sites of distant metastasis, while non-regional lymph node involvement was observed in four patients (28.6%). Detailed patient characteristics and staging information are provided in Table 1 and Table 2.

### 3.2. Disease Management and Outcomes

Surgical resection was performed in all seven patients with Stage I disease—six underwent APR and one underwent local excision. All APR procedures involved sphincter excision and resulted in a permanent end colostomy, in accordance with the standard surgical definition.

Among the patients who underwent surgical resection, four received combined adjuvant radiotherapy and chemotherapy, including interferon. One patient was administered interferon monotherapy as adjuvant treatment, while two patients did not receive any form of adjuvant therapy.

During follow-up, all patients developed disease progression or relapse. The most frequent sites of relapse were the liver (*n* = 12; 86%), non-regional lymph nodes (*n* = 8; 57%), lungs (*n* = 5; 36%), and bones (*n* = 3; 21%).

Chemotherapy and/or immunotherapy response was assessed in all patients. Partial response (PR) was observed in four patients (28.6%), stable disease (SD) in one (7.1%), and progressive disease (PD) in nine (64.3%). Temozolomide was the most commonly used first-line chemotherapy (*n* = 9). No patients received first-line palliative immunotherapy. However, seven patients (50%) received immunotherapy in later lines: nivolumab (*n* = 5) and ipilimumab (*n* = 2). Four patients received third-line therapy, and one patient received fourth-line therapy. Treatment details are provided in Table 3.

**Figure 1:**
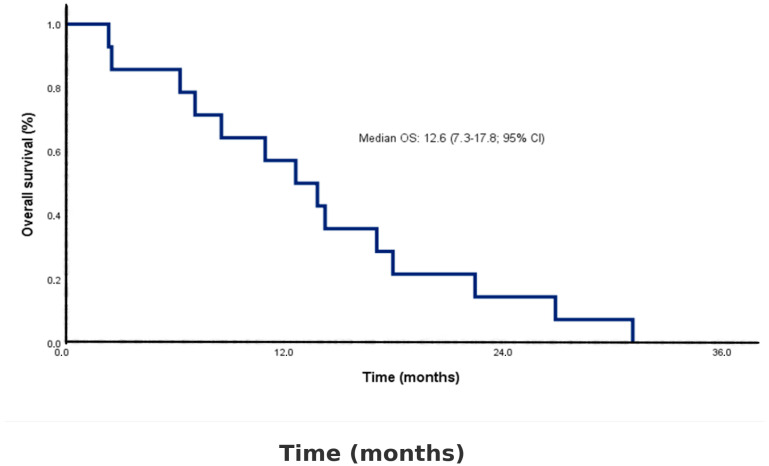
Kaplan–Meier curve for overall survival in the study cohort (*n* = 14). The median overall survival (OS) was 12.6 months (95% confidence interval [CI]: 7.3–17.8). Censored observations are indicated by tick marks on the survival curve.

After a median follow-up of 13.3 months (range: 2.4–31.1 months; 95% CI), the one-year survival rate was 57%, and the two-year survival rate was 14%. The median OS was 12.6 months (95% CI: 7.3–17.8) (Figure 1).

**Figure 2:**
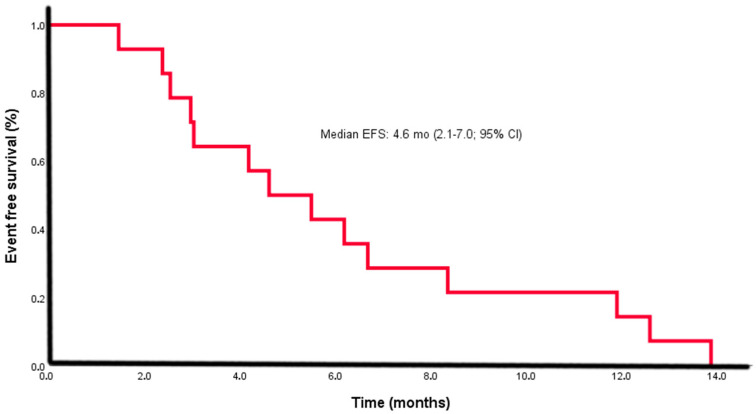
Kaplan–Meier curve for event-free survival (EFS) in the study cohort (*n* = 14). The median EFS was 4.6 months (95% confidence interval [CI]: 2.1–7.0). Censored observations are indicated by tick marks on the survival curve.

All patients experienced relapse or progression within a median of 4.6 months (95% CI: 2.1–7.0) (Figure 2) and ultimately died due to disease progression.

Univariate analysis was conducted to identify potential prognostic factors, including sex, age (<58 vs. ≥58 years), ECOG PS, tumor site (anorectal junction vs. other), endoscopic appearance, disease stage (Stage I vs. Stage II + III), liver metastasis, ulceration, pigmentation, and ostomy status. Male sex (*p* = 0.008) and liver metastasis (*p* = 0.06) were associated with poorer survival.

These two variables, along with tumor site (*p* = 0.168), were included in a multivariate Cox proportional hazards model; however, none were found to be independent predictors of OS. Details are provided in Table 4.

## 4. Discussion

### 4.1. Clinical Features and Diagnostic Challenges

Previous studies on ARMM are predominantly derived from single-center experiences and often present heterogeneous findings. Many of these reports are further limited by short follow-up periods and small sample sizes, making it difficult to draw definitive conclusions regarding prognostic factors. In a 2020 review of 360 ARMM-related publications, 168 included fewer than ten cases, 54 were not specific to ARMM, and another 54 were reviews or opinion papers [16]. Between 1986 and 2019, a total of 144 extracutaneous gastrointestinal tract melanomas were reported from Türkiye, of which 125 were ARMM cases [17]. Within this context, our cohort of 14 well-documented patients, analyzed with systematic survival and prognostic assessments, represents one of the largest single-institution ARMM series from Türkiye and provides a modest yet meaningful addition to the literature. Although diagnostic delays are not unique to ARMM, its extreme rarity, frequent amelanotic histology, and nonspecific presentation substantially increase the risk of initial misdiagnosis. Guided by the principle that “we cannot find what we are not looking for,” a central aim of this study was to underscore that ARMM is often omitted from the differential diagnosis of colorectal diseases—particularly by endoscopists and pathologists—due to its uncommon occurrence and scarce representation in guidelines or textbooks. By contributing our series, we seek to enhance clinical awareness, foster earlier recognition, and promote more accurate diagnosis in future cases.

In our study, the median age at diagnosis was 58 years, consistent with the literature. However, the male-to-female ratio (1.33) contrasted with the female predominance reported in prior studies [17,18,19,20,21,22,23]. This discrepancy might be due to ethnic or genetic differences across populations. The most frequent presenting symptoms were rectal bleeding, changes in bowel habits, and perianal pain—symptoms that are often misattributed to benign conditions such as hemorrhoids or polyps [16,17,18,19,20,21,22,23,24,25]. As in other single-center ARMM series, our cohort shows variability in presentation, diagnostic work-up, and treatment, reflecting real-world practice rather than a uniform protocol.

Accurate tumor localization in ARMM remains difficult due to inconsistent definitions of the anal, perianal, and rectal regions. For example, Memorial Sloan-Kettering Cancer Center reported 24% of ARMM cases as rectal melanomas [25], and a multicenter study involving 640 patients reported 41.4% [2]. A larger dataset of 872 patients indicated the anal region as the most common site (50%), followed by the rectum (34%) [24]. In our series, anorectal junction and anal canal involvement were equally observed (42.9% each), while rectal tumors were less common (14.3%). Such variation could be attributed to environmental, dietary, or hereditary factors.

The diagnostic challenge posed by ARMM cannot be overstated. In our cohort, 42.9% of patients were initially misdiagnosed, resulting in a median diagnostic delay of 5.3 months. Several large series have documented higher misdiagnosis rates in ARMM compared to colorectal adenocarcinoma, often exceeding 40–50%, primarily due to the extreme rarity of the disease, frequent amelanotic histology, and nonspecific symptoms that mimic benign anorectal conditions [13]. This finding aligns with previously published data. Garg et al. [22] reported a misdiagnosis rate of 25.9% among 27 patients, while Falch et al. [13] conducted a comprehensive review of 2652 cases and documented misdiagnosis rates as high as 55%, with diagnostic delays ranging from 3 to 48 months. Similarly, Dodds et al. [5] noted that only 2% of patients were diagnosed at Stage I, underscoring the frequency of late recognition. In the study by Zhang et al. [26], 58.2% of patients were initially misdiagnosed, most commonly with benign anorectal conditions such as hemorrhoids and polyps. Although misdiagnosis alone was not statistically associated with worse overall survival (*p* > 0.05), patients misdiagnosed specifically with hemorrhoids had a significantly poorer prognosis (median OS: 6.0 months, *p* = 0.009).

One major contributing factor is the predominance of amelanotic tumors, which complicates histopathologic identification and increases the likelihood of incorrect or delayed diagnoses. In cutaneous melanoma, pigmentation status is routinely documented in pathology reports and contributes to clinicopathologic characterization. A similar approach should be adopted for ARMM, as consistent reporting of pigmentation status may provide additional insights into its prognostic and diagnostic relevance. Kolosov et al. [21] and Matsuda et al. [10] highlighted how the absence of pigmentation and the overlap of histologic features with other malignancies further hinder timely recognition. Even in large registry-based studies such as the SEER analysis by Chen et al. [2], delayed diagnosis is reflected in the variable distribution of disease stages at presentation. Collectively, our findings reinforce the notion that diagnostic delay in ARMM is not incidental but represents a recurring and systemic challenge in clinical practice.

### 4.2. Treatment Outcomes and Prognostic Factors

For ARMM, surgery remains the foundation of curative-intent management. Unlike cutaneous melanoma, ARMM lacks standardized treatment algorithms, and its resistance to radiotherapy and conventional chemotherapy is well documented [4,8,14,15,16,27,28,29]. Surgery should be considered as only one component of a multidisciplinary management strategy for ARMM, which frequently involves systemic therapy and, in selected cases, radiotherapy. Historical data from Memorial Sloan Kettering Cancer Center demonstrated a shift in primary surgical approach—from routine abdominoperineal resection (APR) in the 1970s and 1980s to wide local excision (WLE)—driven by comparable survival outcomes but lower morbidity with WLE. Furthermore, a substantial proportion of patients present with occult metastases at diagnosis, further underscoring the need for combined-modality treatment [2,4,25]. In our cohort, radical surgery was the primary treatment; wide local excision with ≥1 mm negative margins is preferred when feasible, as abdominoperineal resection offers no proven survival advantage and is associated with higher morbidity, and it is thus reserved for palliation or salvage. Adjuvant radiotherapy was administered in some cases with close or positive margins to improve local control, but no survival benefit was observed. All patients eventually developed distant metastases, underscoring the need for more effective systemic therapy. Over the past decade, immune checkpoint inhibitors—anti-programmed cell death-1 (anti–PD-1) antibodies (nivolumab, pembrolizumab) and anti-cytotoxic T-lymphocyte-associated antigen 4 (anti–CTLA-4) antibodies (ipilimumab)—have transformed melanoma care, with targeted therapies available for select patients with BRAF or KIT mutations. Although prospective data in mucosal melanoma, including ARMM, are scarce, retrospective series suggest meaningful activity [8,14,15,16]. In our study, no patients received first-line immunotherapy, reflecting historical practice patterns; however, 50% later received checkpoint inhibitors, highlighting a growing shift toward immunotherapy even in rare mucosal subtypes. Combination ipilimumab–nivolumab may achieve higher response rates than anti-PD-1 monotherapy in mucosal melanoma, albeit with greater toxicity. Given ARMM’s aggressive biology and poor prognosis, earlier integration of immunotherapy into multimodal treatment for locally advanced or metastatic disease warrants further prospective evaluation to optimize sequencing and identify predictive biomarkers [8,14,15,16].

Our cohort showed a median OS of 12.6 months (95% CI: 7.3–17.8) with a 1-year survival rate of 57% and 2-year survival of 14%, which aligns with published survival ranges for ARMM (8–19 months) [2,4,10,16,30,31]. Previous Turkish studies reported median OS ranging from 8.7 to 16 months [6,16,17,18,19,20]. All patients experienced a relapse or progression within a median interval of 4.6 months (2.1–7.0 months; 95% CI). Thus, our outcomes appear consistent with national and international data.

Univariate analysis identified male sex and liver metastasis as significant predictors of poor OS. Median OS was 7.8 months for males vs. 20.2 months for females and 6.2 months in those with liver metastases vs. 17.1 months without. However, neither remained statistically significant in multivariate analysis, likely due to the limited sample size. The observed numerical trends—particularly for male sex, liver metastasis, and tumor location—may still be clinically relevant. These findings warrant validation in larger, multicenter studies to better define their prognostic implications. Prior studies have shown mixed results regarding gender and OS [21,24,25,30].

Although we observed a numerical OS difference based on tumor site (6.7 months for anorectal junction vs. 17.5 months for rectal/anal tumors), statistical significance was not reached. Similarly, Stage I patients had twice the OS of Stage II–III patients, but this was not statistically significant either. Other studies have reported tumor stage as an independent predictor of OS [6,31]. In one study, early-stage patients had an OS of 18 months versus 11 months in metastatic cases [19], while Ballantine Stage III was associated with poor prognosis in another [22].

### 4.3. Limitations

This study has several important limitations. First, its retrospective design restricted access to detailed histopathological variables such as tumor thickness, depth of invasion, perineural and lymphovascular invasion, and surgical margin status—all of which are established prognostic indicators. Additionally, data on alternative outcome measures, including cancer-specific survival, were not available. Second, the small sample size (n = 14) limited the statistical power to detect significant associations and precluded robust subgroup analyses. Although multivariate Cox regression was performed using clinically relevant variables, the wide confidence intervals reflected imprecision arising from the low number of events. Moreover, the limited use of immunotherapy during the earlier years of this study hindered a comprehensive evaluation of its therapeutic impact. Despite these methodological constraints, this study provides valuable real-world insight into a rare malignancy with limited high-quality data.

## 5. Conclusions

ARMM is a rare and aggressive malignancy, often diagnosed at advanced stages due to nonspecific symptoms and frequent misdiagnosis. In our cohort, 42.9% of patients experienced a median diagnostic delay of 5.3 months. To minimize such delays, clinicians should promptly biopsy any persistent or atypical anorectal lesion, incorporate melanocytic immunohistochemical staining (S-100, HMB-45, Melan-A) into the diagnostic work-up, and consider early use of advanced imaging (MRI or PET-CT) when findings are suspicious or inconclusive. These measures should be implemented even when the initial presentation resembles benign conditions. Although our sample size was limited, tumor location and liver metastasis appeared to have potential prognostic relevance, warranting confirmation in larger, multicenter studies. By documenting diagnostic delays, providing survival and prognostic analyses from one of the largest single-center Turkish cohorts, this study underscores that in rare cancers like ARMM, timely recognition and early intervention can be decisive in achieving curative outcomes.

## Figures and Tables

**Table 1:** Baseline characteristics of the study cohort (*n* = 14).

Characteristics	*n* (%)
**Age (years)** *	58 ± 12
**Gender**	
**Male**	8 (57.1)
**Female**	6 (42.9)
**Tumor anatomic site**	
**Rectal**	2 (14.3)
**Anorectal**	6 (42.9)
**Anal canal**	6 (42.9)
**Presentation symptoms**	
**Rectal bleeding**	10 (71.4)
**Weight loss**	6 (42.9)
**Anorectal mass**	5 (35.7)
**Change in bowel habits**	5 (35.7)
**Perianal pain**	2 (14.3)
**Misdiagnosis**	
**No**	8 (57.1)
**Hemorrhoids**	2 (14.3)
**Polyp**	1 (7.1)
**Normal colonoscopic findings**	3 (21.4)
**Endoscopic evaluation of tumor**	
**Polypoid**	5 (35.7)
**Luminal mass**	9 (64.3)
**Ulceration**	
**Yes**	4 (28.6)
**No**	4 (28.6)
**Unknown**	6 (42.9)
**Pigmentation**	
**Melanotic**	3 (21.4)
**Amelanotic**	2 (14.3)
**Unknown**	9 (64.3)
**Lymphovascular invasion**	
**Yes**	3 (21.4)
**No**	2 (14.3)
**Unknown**	2 (14.3)
**BRAF mutation**	
**Negative**	12 (85.7)
**Mutated**	0 (0)
**Unknown**	2 (14.3)

* Age is presented as mean ± standard deviation; other variables are shown as number and percentage.

**Table 2:** Staging and metastasis status at diagnosis (*n* = 14).

Characteristics	*n* (%)
**Stage at diagnosis**	
**Localized (Stage I)**	7 (50.0)
**Regional (Stage II)**	1 (7.1)
**Distant (Stage III)**	6 (42.9)
**Distant metastasis pattern**	
**Liver**	1 (7.1)
**Liver and bone**	1 (7.1)
**Liver and lung**	2 (14.3)
**Liver and lymph node**	1 (7.1)
**Lung and lymph node**	1 (7.1)
**Lymph node**	1 (7.1)
**Non-regional lymph node metastasis**	
**Yes**	4 (28.6)
**No**	10 (71.4)
**Metastasis to liver**	
**Yes**	5 (35.7)
**No**	9 (64.3)

Data are presented as number (percentage). Percentages are calculated based on the total number of patients with available data for each variable.

**Table 3:** Surgical and medical treatments (*n* = 14).

Characteristics	*n* (%)
**Initial treatment modality**	
**Surgery**	2 (14.3)
**Surgery + adjuvant therapy**	5 (35.7)
**Palliative chemotherapy**	7 (50.0)
Type of surgery *	
**Abdominoperineal resection**	6 (85.7)
**Local excision**	1 (14.3)
Adjuvant interferon *	
**Yes**	5 (71.4)
**No**	2 (28.6)
Adjuvant radiotherapy *	
**Yes**	4 (57.1)
**No**	3 (42.9)
**Ostomy status**	
**Yes**	5 (35.7)
**No**	9 (64.3)
**First-line palliative chemotherapy**	
**Temozolomide**	9 (64.3)
**DTIC + Carboplatin**	4 (28.6)
**Paclitaxel + Carboplatin**	1 (7.1)
**Second-line palliative therapy ^†^**	
**Temozolomide**	2 (22.2)
**Nivolumab**	5 (55.6)
**Ipilimumab**	2 (22.2)
**Third-line palliative therapy ^‡^**	
**Temozolomide**	1 (25.0)
**Paclitaxel + Carboplatin**	1 (25.0)
**DTIC + Ipilimumab**	2 (50.0)
**Fourth-line palliative therapy**	
**DTIC + Ipilimumab**	1 (100)
**Outcome**	
**Alive**	0 (0)
**Deceased**	14 (100)
**Follow-up time, median (range), months**	13.3 (2.4–31.1)
**Time to event (relapse/progression), median (IQR), months (EFS)**	4.6 (2.1–7.0)
**Time to death, median (IQR), months (OS)**	12.6 (7.3–17.8)

Data are presented as number (percentage) unless otherwise specified. Percentages are calculated based on the total number of patients with available data for each variable. * Percentage calculated among patients undergoing surgery. ^†^ Percentage calculated among patients receiving second-line therapy. ^‡^ Percentage calculated among patients receiving third-line therapy. **DTIC:** dacarbazine.

**Table 4:** Univariate and multivariate Cox proportional hazards model of overall survival.

Variable	*n* (%)	Median OS (95% CI), Months	Univariate Analysis HR (95% CI), *p*-Value	Multivariate Analysis HR (95% CI), *p*-Value
**Sex**				
**Male**	8 (57.1)	7.8 (2.4–14.2)	0.056 (0.01–0.48), 0.008	0.11 (0.01–1.23), 0.07
**Female**	6 (42.9)	20.2 (14.1–31.1)	—	—
**Age at diagnosis**				
**<58 years**	7 (50.0)	12.6 (2.4–22.4)	1.86 (0.58–5.99), 0.293	—
**≥58 years**	7 (50.0)	14.2 (2.5–31.1)	—	—
**ECOG PS**				
**0**	8 (57.1)	14.2 (8.0–20.3)	0.98 (0.31–3.10), 0.976	—
**1**	6 (42.9)	7.0 (4.3–9.8)	—	—
**Tumor site**				
**Anorectal**	6 (42.9)	6.7 (2.4–11.0)	0.34 (0.08–1.36), 0.168	0.86 (0.01–11.03), 0.936
**Rectum/anal canal**	8 (57.1)	17.5 (12.6–31.1)	—	—
**Endoscopic evaluation**				
**Polypoid**	5 (35.7)	14.2 (12.6–18.1)	1.00 (0.29–3.38), 0.99	—
**Luminal mass**	9 (64.3)	8.5 (2.4–31.1)	—	—
**Stage**				
**Stage I**	7 (50.0)	14.2 (13.1–15.3)	1.39 (0.46–4.22), 0.55	—
**Stage II–III**	7 (50.0)	7.0 (4.9–9.2)	—	—
**Liver metastasis**				
**Yes**	9 (64.3)	6.2 (2.4–11.0)	2.24 (0.48–10.25), 0.06	1.68 (0.17–16.12), 0.65
**No**	5 (35.7)	17.1 (8.5–31.1)	—	—
**Pigmentation**				
**Melanotic**	3 (21.4)	6.3 (2.4–22.4)	0.73 (0.38–1.42), 0.364	—
**Amelanotic**	2 (14.3)	13.3 (12.6–14.1)	—	—
**Unknown**	9 (64.3)	14.2 (2.5–31.1)	—	—
**Ulceration**				
**Yes**	4 (28.6)	15.6 (7.1–22.4)	1.01 (0.53–1.92), 0.96	—
**No**	4 (28.6)	7.4 (2.5–31.1)	—	—
**Unknown**	6 (42.9)	13.4 (2.4–26.8)	—	—
**Ostomy**				
**Yes**	5 (35.7)	12.6 (6.3–22.4)	1.52 (0.47–4.98), 0.48	—
**No**	9 (64.3)	14.2 (2.4–31.1)	—	—

OS: overall survival; ECOG PS: Eastern Cooperative Oncology Group performance status; HR: hazard ratio; CI: confidence interval. HR and *p*-values are from Cox proportional hazards models. Dashes (—) indicate reference categories.

## Data Availability

The data presented in this study are available from the corresponding author upon reasonable request. Due to institutional ethical regulations and patient confidentiality, the datasets are not publicly accessible.
